# Parent-Reported Outcomes of Early Childhood Selective Dorsal Rhizotomy for the Treatment of Spastic Diplegia

**DOI:** 10.7759/cureus.15530

**Published:** 2021-06-08

**Authors:** TS Park, Susan Joh, Deanna M Walter, Nicole L Meyer

**Affiliations:** 1 Pediatric Neurosurgery, Washington University School of Medicine, St. Louis Children's Hospital, St. Louis, USA

**Keywords:** children, early selective dorsal rhizotomy, spastic diplegia, spasticity, quality of life, pediatric orthopedic surgery

## Abstract

Background

A selective dorsal rhizotomy (SDR) is employed to treat spastic cerebral palsy. The surgical techniques and patient care protocols vary among hospitals. One of the variations is the age cut-off for SDR. We have been advocating SDR to be performed early - especially at ages 2 and 3. With this study, we are reporting the feasibility and parent-reported surgical outcomes of receiving SDR at an early age for the treatment of spastic diplegia.

Objectives

Our aim is to examine the safety and benefits of receiving SDR at the ages of 2 and 3 for the treatment of spastic diplegia.

Methods

The Institutional Review Board (IRB) of Washington University School of Medicine approved this retrospective quality of life survey and chart review (approval #202009056). The subjects of this study were children and teens (ages: 3.9-18.1) with spastic diplegic cerebral palsy who underwent SDR at ages 2 or 3 between years 2005 and 2019 at St. Louis Children’s Hospital. Only domestic patients that were minors at the time of the study were selected to be participants in compliance with IRB regulations to protect patient health information that could potentially be breached by sending information to an incorrect or dated email. Thus, all contact was made through postal mail. The study included 141 patients from a total of 362 eligible patients. Parents of eligible patients were sent the research survey via postal mail. Only patients who responded to the survey were included in this study. The survey included questions on demographic information, quality of life, health perception, motor and ambulatory functions, braces and orthotics, pain issues, side effects of SDR, and post-SDR treatment.

Results

The study included 141 diplegic patients. Of all patients at the time of the study, 91% reported an improvement in walking, 92% in standing, and 89% in sitting. In daily life activities, 87% of patients became more independent after SDR. 65% of patients were able to walk without a walking aid and about 4% were not able to walk. 11% of all patients relied mostly on a wheelchair. Moreover, 43% of patients were able to run independently. Regarding post-SDR orthopedic surgery, 48% of patients received at least one type of orthopedic surgery, with Achilles tendon lengthening, hamstring lengthening, and calf muscle release being the most common types.

Conclusions

SDR performed at an early age through a single-level laminectomy was proved feasible and safe. A follow-up until the adult age (18 years) showed improvements in walking and other motor functions. The results support the implementation of early-age SDR for the treatment of spastic diplegia.

## Introduction

Selective dorsal rhizotomy (SDR) is employed around the world to treat spastic cerebral palsy (CP). The surgical techniques of SDR and overall patient care methods have evolved over the last three decades [1-5]. Individual centers have adopted different protocols in their practices, with age cut-offs for SDR being one of the differences. In St. Louis Children’s Hospital, we have been advocating receiving SDR at an early age (2 and 3 years) for many years. We report the feasibility of early-age SDR and parent-reported surgical outcomes for the treatment of spastic diplegia in this study.

## Materials and methods

The Institutional Review Board (IRB) of Washington University School of Medicine approved this retrospective quality of life survey and chart review (approval #202009056). The subjects of this study were children and teens (ages: 3.9-18.1) with spastic diplegic CP who underwent SDR at ages 2 or 3 (up to the fourth birthday) between years 2005 and 2019 at St. Louis Children’s Hospital. Only domestic patients who were minors at the time of the study were selected to be participants in compliance with IRB regulations to protect patient health information that could potentially be breached by sending information to an incorrect or dated email. Therefore without prior in-person consent to email patients for research recruitment, all contact was made through postal mail. International patients were not recruited as using postal mail with these patients was not possible; we did not have their full address. We gathered contact information from our clinic’s database and medical records, which only includes the mailing addresses of the patients’ parents or guardians. Only minors were recruited because parents and guardians are fully able to consent for a minor and access a minor’s health information. Consent was obtained from the patients’ guardians by filling out the survey and sending it back.

The survey was constructed with questions on demographic information, perception of health, quality of life, pre and postoperative motor and ambulatory functions, post-SDR treatment, braces and orthotics, side effects of SDR, and perception of the SDR procedure. Demographic information included the date of birth, living situation, and current student and employment status.

A five-point scale from poor to excellent was utilized to evaluate the perception of the patients’ health, as seen in Question 1 of the SF-36 health survey. Yes/no questions were used to indicate improvement due to SDR in abilities in walking, standing, sitting, ability to exercise, ability to stretch, and endurance.

Yes/no questions were used to evaluate the quality of life. Questions included receiving help with eating, hygiene, bladder, and transferring positions. Questions were asked about the patients’ independence after SDR, engagement in recreational sports, and regularity of strengthening and stretching muscles.

The Gross Motor Function Classification Scale (GMFCS) was used to construct questions assessing patients’ pre- and post-SDR motor function including running, walking, and sitting. Different questions were used for different age groups (2-3, 4-5, 6-11, and 12-18 years) for the differences in each stage of motor development. Separate questions were included to assess pre- and post-SDR ambulatory function by asking patients to indicate their level of function based on a five-level classification system. Level 1 includes patients who can run independently and level 5 includes patients who cannot walk.

Questions about bladder function, pain, and loss of sensation in the lower limbs were asked to assess the side effects of SDR. Participants who indicated numbness or loss of sensation were asked to further indicate the location of the numbness. Patients were asked to provide any other negative side effects if present. Post-SDR questions about scoliosis, post-SDR orthopedic surgical interventions, medications for spasticity, and Botox were also inquired.

Yes/no/unsure questions were used to ask patients if they had benefited from SDR and if they would recommend the procedure to other patients. They were also given the option to add any additional comments about their perception of SDR.

## Results

Study cohort

The total number of patients diagnosed with diplegic CP who received an SDR at ages 2 and 3 (up to the fourth birthday) during the study period (2005-2019) is 744 patients. Participants chosen for this research study included only domestic patients who were minors and followed after SDR for at least one year; international patients and those above the age of 18 years were excluded in compliance to IRB regulations to protect patient health information that could be breached by sending information to an incorrect email. The IRB required that all patients be contacted only through postal mail and not email as we did not have prior in-person consent to email them specifically for research recruitment. The research team identified patients who qualified for the study - domestic patients with updated residential addresses - and gathered contact information from the Center for Cerebral Palsy Spasticity at St. Louis Children’s Hospital’s database. Of the 744 patients, we found a total of 362 eligible patients and received 141 responses, resulting in a 39% response rate. All patients underwent a single-level laminectomy SDR as described in an earlier publication [6].

Demographics of the study cohort

The participants’ ages were 2.1 to 4.0 years (mean age: 3.1 ± 0.5) at the time of surgery (Table 1). When they completed the survey, the patients were 3.9 to 18.1 years (mean age: 9.7 ± 4.0). All ages are considered the patient’s current age (i.e. age 5.9 is 5 years old). The follow-up period, referring to the period between the date of surgery and the date of the follow-up study, ranged from 1.0 to 15.2 years. In that, 57% of participants identified as male and 43% identified as female and 97% of patients were attending school at the time of the survey.

The 3% of patients who were not attending school were 5 years or younger. However, 25 patients that were 5 years or younger at the time of completing the survey were attending school. Eleven patients (ages: 3-10) were attending school part-time. This survey took place during the COVID-19 pandemic and may have affected this data. Four percent of patients were working, all part-time (ages: 14-17).

**Table 1:** Demographics of 141 diplegic patients at the time of the survey

Study Population	
Total No. of Patients	141 patients
Age at surgery	2.1–4.0 years (3.1 ± 0.5, mean ± SD)
Age at follow-up survey	3.9–18.1 years (9.7 ± 4.0, mean ± SD)
Follow-up period	1.0–15.2 years (6.5 ± 4.0, mean ± SD)
Sex	% of patients
Male	57
Female	43
Education	% of patients
Currently attending school	97

Health perception after SDR

Almost all patients (96%) reported a health perception of good or better. In that, 44% of patients reported “Excellent”, 45% reported “Very good”, and 7% reported “Good”. Only 1% reported “Fair” and none reported “Poor”. Three patients had no answer for health perception, and two of those patients had no answers for the entire section (including improvements) (Table 2).

A majority of the patients reported improvements in each of the categories questioned: walking (91%), standing (92%), sitting (89%), balance when walking (79%), ability to exercise (86%), ability to stretch (92%), endurance (79%), and recreational sports (60%). Most frequent improvements were seen in the ability to stretch, stand, and walk. Not every patient had answers to every question.

**Table 2:** Health and physical ability improvement perception after SDR of 141 diplegic patients *Not all patients responded to all questions.

Health Perception	% of patients		
Excellent	44		
Very good	45		
Good	7		
Fair	1		
Poor	0		
No response	2		
Improved Motor Functions*			
	Yes (%)	No (%)	Unsure (%)
Walking	91	6	1
Standing	92	6	0
Sitting	89	9	1
Balance when walking	79	16	1
Ability to exercise	86	11	1
Ability to stretch	92	6	0
Endurance	79	17	1
Recreational sports	60	33	0

Independence in daily physical activities

Patients were separated into age groups as children typically require help in several physical activities when they are younger. Thirty patients (21%) were aged 4 to 5, 61 patients (43%) were aged 6 to 10, and 50 patients (36%) were aged 11 to 18 (Table 3).

Patients aged 4 and 5 were not included in this section because children in this age group typically require help in several aspects of daily life. Children typically begin to become more independent at age 6.

For patients aged 6 to 10, one patient had no answers to all questions in this section. One patient (1% overall) indicated that they sometimes have help getting dressed and using the toilet. This patient was included with others that answered “yes” to requiring help with those activities.

For patients aged 11 to 18, one patient had no answer for requiring help during eating, and another had no answer for requiring help getting dressed.

From the data, a general trend of more independence in daily activities can be seen, yet this trend is likely due to normal aging as all patients are at least a year post-treatment. To account for this possibility, questions were asked about improvement in independence after SDR. 87% of all patients reported improvement in independence overall, 11% reported no change, and 2% did not answer. One patient who had no answer commented that improvement in independence was hard to judge as the improvements may have been from the natural course of growing up. Two patients believed their course of ability to walk worsened after SDR.

**Table 3:** Independence in daily physical activities by age group of 141 diplegic patients *Percentage is out of the number of patients in the categorized age group. **30 patients aged 4-5 were left out as most patients required help in all activities because of age.

Daily Activities				
Requires:	% of patients* **			
	Ages 6-10		Ages 11-18	
(# of patients)	61 patients		50 patients	
	Yes	No	Yes	No
Help eating	11	87	10	88
Help using the toilet	38	61	28	72
Help getting dressed	59	39	32	66
Exercises regularly	93	3	78	22
Stretches legs regularly	80	18	66	34
Plays recreational sports	33	66	34	66

Gross motor functions before and after SDR

Different age groups were asked different questions about gross motor functions according to their developmental stage. Questions constructed for this section were based on the Gross Motor Function Classification System (GMFCS) [7]. Pre-SDR gross motor function questions were based on abilities typical for 2 and 3-year-olds. Before SDR, 55% of patients preferred creeping and crawling, 57% cruised while holding onto furniture, and 46% required help steering when walking. For sitting ability, 45% of patients stood and sat without help from an adult (Table 4).

Post-SDR questions were more similar across age groups as children should be able to walk by age 4; therefore, patients were not separated into age groups for this section. In that, 65% of patients were able to walk without a walking aid and 32% were able to walk with a walking aid, totaling 97% that can walk (Table 4). However, 4% of patients indicated that they do not walk (Table 5). Of the patients that do walk, 39% indicated that they can walk on all surfaces and 57% have difficulty walking on uneven surfaces. Forty-six percent of patients use a wheelchair for long distances or outside including 11% of all patients that mostly rely on a wheelchair. Also, 92% can sit and stand without help (previously 45%) (Table 4).

GMFCS is a classification system in which the gross motor function of children and teens with CP are categorized into five different levels. Level I is the highest functioning category and level V is the lowest. Pre-SDR GMFCS levels were found from our physical therapy records. Post-op levels were also found from physical therapy records but were mostly determined by the survey responses as the survey was the most recent measure of gross motor function. All 26% of patients with a pre-op GMFCS level I also had a post-SDR GMFCS of level I; 45% of patients classified as level I postoperatively. All patients were at least level IV both before and after SDR; 12% were level IV before the surgery and only 9% were level IV after (Table 4).

**Table 4:** Gross motor functions in different age groups of 141 diplegia patients before and after SDR Gross motor functions with a dash (-) were those not applicable to the age group and therefore were not asked. *Four percent of patients do not walk.

Gross Motor Functions	% of patients	
	Pre-SDR	Post-SDR
Walking		
Prefers creeping and crawling	55	-
Cruises holding furniture	57	-
Help steering when walking	46	-
Walks (no walking aid)	-	65
Walks only with walking aid	-	32
Can use stairs (no handrails)	-	23
Able to walk on all surfaces	-	39*
Difficulty walking on uneven surfaces	-	57*
Wheelchair for long distances or outside	-	46
Sitting		
Stands and sits (no help)	45	92
Wheelchair (home, school, community)	-	11
GMFCS Level		
I	26	45
II	33	23
III	30	23
IV	12	9

**Table 5:** Ambulatory function of 141 diplegic patients before and after SDR

Ambulatory Function	% of patients	
	Pre-SDR	Post-SDR
Walks without mobility device in all environments	14	52
Walks without mobility device in protected environments	13	13
Walks with crutches or canes	3	15
Walks with walker	52	16
Does not walk	18	4
Running		
Runs independently	3	43

Ambulatory function after SDR

SDR procedure proved to be beneficial to many patients (Table 5). All patients who could run independently before the procedure were able to run independently after. Fourteen patients walked without a mobility device in all environments, 14 patients walked without a mobility device in protected environments, 21 patients walked with a walker, three patients walked with crutches or canes, and four patients did not walk could run independently after. It is important to note that some of the children in this study were under 2 years old and all were at most age 4.0 before SDR. At this age, children are still developing their ability to walk. For this reason, questions about walking ability were not asked in the previous section. However, because many children start walking at age 2, questions were asked about ambulatory function before and after SDR to compare the change in ability.

All but one patient that was not able to walk before SDR could not walk after. The patient that could not walk after the procedure indicated that they walked with a walker before SDR.

Orthopedic surgery after SDR

Orthopedic surgery is a crucial procedure for patients with spastic CP with muscle contractures and is performed on patients to release muscle tightness. SDR allows for these patients to have a minimally invasive muscle-tendon lengthening orthopedic procedure as it reduces spasticity. For this patient cohort, 48% of patients reported receiving orthopedic surgery; 29% had only one type of orthopedic surgery, and 18% had two or more types. Two patients indicated receiving orthopedic surgery but did not specify the type of surgery. Achilles tendon lengthening (21%), hamstring lengthening (18%), and calf muscle release (16%) were the most common orthopedic surgeries (Table 6). It is important to note that the need for orthopedic surgery is not a result of SDR. The high incidence of orthopedic surgery is due in part to our practice preference for performing early orthopedic surgery in children 5 years old and older. Another reason is the fact that due to motor impairment, many diplegic children have limited knee extension and foot dorsiflexion while walking. As a result, the hamstrings and calf muscles are not able to fully stretch during daily activities, and muscle contractures develop even without spasticity. The restricted knee extension and foot dorsiflexion may persist even after SDR causing limited hamstring and calf muscle flexibility when walking and participating in other physical activities, thus requiring orthopedic intervention. With this in mind, monitoring children closely after SDR to detect early deformities is essential.

A small percentage of patients (6%) were diagnosed with scoliosis. One indicated that the scoliosis was very minor and another that they were on the “watch list” but their physician was pleased. Only one patient received a spine fusion for their scoliosis (Table 6).

**Table 6:** Treatment after SDR in 141 diplegic patients *Each patient may have had more than one type of surgery.

Treatment	% of patients
Orthopedic surgery*	
Adductor release	7
Hamstring lengthening	18
Calf muscle release	16
Achilles tendon lengthening	21
Hip surgery	9
Spine fusion for scoliosis	1
Current Medications	
Baclofen	4
Botox	1

## Discussion

This is a retrospective non-controlled study in which data were collected using a survey from a portion of the potential study cohort. The study was conducted under the new constraining rules of the IRB. We were barred from contacting patients’ parents by email and were required to contact parents only by postal mail. This limitation required us to exclude international patients and many domestic patients as we did not have postal addresses for these patients. We were also restricted to mail only minors, as they are under the protection of their parents or guardian, meaning that patient-protected information is accessible to parents and guardians. Thus, we were required to exclude patients who reached adult age. Notwithstanding the unprecedented limitations, we analyzed the feasibility, safety, potential benefits, and adverse effects of early childhood SDR. We expect that the results of our study will expand the strength of clinical experience with SDR for the treatment of spastic diplegia in children.

Most importantly, we provide evidence that the single-level laminectomy SDR on 2 and 3-year-old children are feasible and safe. In the study participants, we did not encounter postoperative complications such as a spinal fluid leak, paralysis, urinary incontinence, or surgical site infection requiring intravenous antibiotics. In patients who are near the end of childhood, we found no cases of SDR-related spinal problems or significant sensory loss. Likewise, none of the patients experienced a return of spasticity. Based on the results of an earlier study conducted on adult patients who had SDR in childhood [2,3], we are expecting that the safety of SDR will hold up later in adult life.

In able-bodied children, a mature gait pattern is established by 3 years of age, and their adult gait pattern is established by 7 years [8]. Beyond age 7, motor coordination is further refined by practice. Children with spastic CP are born with normal muscles. In late infancy, spasticity becomes symptomatic and begins to exert negative effects on the muscles. Spasticity damages muscles; it also decreases voluntary movements and associated muscle flexibility. Spasticity inhibits longitudinal muscle growth [9-11]. Muscle contractures and bony deformities develop due to the effects of spasticity and reduced active movements [12]. It is thus possible that early intervention of reducing spasticity with SDR would be more beneficial than late intervention.

Furthermore, we found that after 4 years of age, non-surgical treatment cannot reverse the established muscle contractures, and orthopedic surgery becomes the only treatment option. However, removing the patient's spasticity through SDR enables a minimally invasive muscle-tendon lengthening orthopedic surgery to treat the contractures [13]. It is noteworthy that nearly half of the participants in our study received orthopedic surgery despite early-age SDR. The high incidence of orthopedic surgery is due in part to our practice preference for performing early orthopedic surgery in children ages 5 and older. Another reason is the fact that due to motor impairment, many diplegic children have limited knee extension and foot dorsiflexion while walking. As a result, the hamstrings and calf muscles are not able to fully stretch during daily activities, and muscle contractures develop even without spasticity. The restricted knee extension and foot dorsiflexion may persist even after SDR causing limited hamstring and calf muscle flexibility when walking and participating in other physical activities. With this in mind, monitoring children closely after SDR to detect early deformities is essential.

Gross motor function and ambulatory function improved after SDR (Tables 4 and 5). Due to the granular data of each age group and the lack of a control group, we cannot determine the significance of the role of early-age SDR in improving postoperative motor function. Nevertheless, the results are promising in that the percentage of patients who can walk independently increased from 14% pre-surgery to 52% post-surgery, and those able to run increased from 3% to 43%. The improvements observed in overall motor functions and the absence of adverse effects from surgery suggest that early-age SDR for treatment of spastic diplegia is a viable option.

## Conclusions

One hundred forty-one children underwent SDR at 2 and 3 years of age for spastic diplegia. No post-surgical complications were reported in the participants following an early-age SDR performed through a single-level laminectomy, confirming the safety of the procedure. The follow-up surveys of the patients revealed improvements in walking and other motor functions. The results support the intervention of early-age SDR for the treatment of spastic diplegia.
